# miRNA independent hepacivirus variants suggest a strong evolutionary pressure to maintain miR-122 dependence

**DOI:** 10.1371/journal.ppat.1006694

**Published:** 2017-10-30

**Authors:** Yingpu Yu, Troels K. H. Scheel, Joseph M. Luna, Hachung Chung, Eiko Nishiuchi, Margaret A. Scull, Natalia Echeverría, Inna Ricardo-Lax, Amit Kapoor, Ian W. Lipkin, Thomas J. Divers, Douglas F. Antczak, Bud C. Tennant, Charles M. Rice

**Affiliations:** 1 Laboratory of Virology and Infectious Disease, Center for the Study of Hepatitis C, The Rockefeller University, New York, NY, United States of America; 2 Copenhagen Hepatitis C Program, Department of Infectious Diseases, Hvidovre Hospital, and Department of Immunology and Microbiology, Faculty of Health and Medical Sciences, University of Copenhagen, Copenhagen, Denmark; 3 Laboratorio de Virología Molecular, Centro de Investigaciones Nucleares, Facultad de Ciencias, Universidad de la República, Montevideo, Uruguay; 4 Department of Pediatrics, College of Medicine, The Ohio State University, Columbus, OH, United States of America; 5 Center for Vaccines and Immunity, The Research Institute at Nationwide Children’s Hospital, Columbus, OH, United States of America; 6 Center for Infection and Immunity, Mailman School of Public Health and College of Physicians & Surgeons, Columbia University, New York, NY, United States of America; 7 Department of Clinical Sciences, College of Veterinary Medicine, Cornell University, Ithaca, NY, United States of America; 8 Baker Institute for Animal Health, College of Veterinary Medicine, Cornell University, Ithaca, NY, United States of America; University of California, San Diego, UNITED STATES

## Abstract

Hepatitis C virus (HCV) requires the liver specific micro-RNA (miRNA), miR-122, to replicate. This was considered unique among RNA viruses until recent discoveries of HCV-related hepaciviruses prompting the question of a more general miR-122 dependence. Among hepaciviruses, the closest known HCV relative is the equine non-primate hepacivirus (NPHV). Here, we used Argonaute cross-linking immunoprecipitation (AGO-CLIP) to confirm AGO binding to the single predicted miR-122 site in the NPHV 5’UTR *in vivo*. To study miR-122 requirements in the absence of NPHV-permissive cell culture systems, we generated infectious NPHV/HCV chimeric viruses with the 5’ end of NPHV replacing orthologous HCV sequences. These chimeras were viable even in cells lacking miR-122, although miR-122 presence enhanced virus production. No other miRNAs bound this region. By random mutagenesis, we isolated HCV variants partially dependent on miR-122 as well as robustly replicating NPHV/HCV variants completely independent of any miRNAs. These miRNA independent variants even replicate and produce infectious particles in non-hepatic cells after exogenous delivery of apolipoprotein E (ApoE). Our findings suggest that miR-122 independent HCV and NPHV variants have arisen and been sampled during evolution, yet miR-122 dependence has prevailed. We propose that hepaciviruses may use this mechanism to guarantee liver tropism and exploit the tolerogenic liver environment to avoid clearance and promote chronicity.

## Author summary

It has been known for more than 10 years that the hepatitis C virus (HCV) genome binds two copies of the liver-specific microRNA (miRNA), miR-122. But until recently, it was unknown whether this interaction was unique to HCV or also conserved among other hepaciviruses. Now, due to our expanded view of the hepacivirus family, we know that most, if not all, hepaciviruses have at least one predicted miR-122 binding site in their 5’ untranslated region (5’UTR). In this study, we aimed to obtain a broader understanding of hepacivirus/miR-122 interactions and determine how miR-122 dependence influences tissue tropism. To do this, we chose to study the equine non-primate hepacivius (NPHV), the closest relative of HCV. NPHV has one predicted miR-122 site in its 5’UTR. We show that minor changes in the 5’UTR of HCV and NPHV/HCV chimeras weaken or obviate the need for miR-122 for virus replication. Overall, our data suggest that miR-122-independent hepaciviruses have likely been sampled during evolution, but hepaciviruses may have been selected to utilize miR-122 to restrict replication to the tolerogenic liver environment to help avoid immune clearance.

## Introduction

Chronic HCV infection is one of the most common liver diseases with ~71 million people persistently infected globally; a significant number of those will develop cirrhosis or liver cancer [1]. The binding of liver specific miR-122 to HCV RNA is essential for viral replication [2]. This interaction is unusual in that two molecules of miR-122 bind to the 5’ untranslated region (5’UTR) of HCV using both seed and auxiliary pairing [3,4]. It is well established that HCV viral load can be dramatically decreased by inhibiting miR-122 in cell culture, chimpanzees or patients, making antagonists of miR-122 a first-in-class antiviral strategy [5–7]. Several functions have been suggested for the HCV/miR-122 interaction: (i) Binding of the AGO/miR-122 complex can protect the uncapped HCV RNA from degradation by cellular exonuclease XRN1 and/or XRN2 [8–10]. (ii) AGO/miR-122 binding can increase HCV internal ribosome entry site (IRES)-driven translation, thus promoting the HCV replication [11]. This process possibly works by switching the IRES from “closed” to “open” conformation [12,13]. (iii) Competition between miR-122 and poly(rC)-binding protein (PCBP2) that binds and circularizes HCV RNA may act as a switch between translation and replication [14]. (iv) In addition, using AGO-CLIP and RNA-seq, we recently showed that HCV RNA can act as a miR-122 “sponge” in a positive feed-back loop to de-repress cellular mRNAs normally targeted by miR-122, thereby indirectly regulating hundreds of genes [15].

Until recently, the miRNA dependence of HCV was considered unique among viruses, even for viruses possessing similar IRESs. However, we recently demonstrated that pestiviruses, such as the important veterinary pathogens bovine viral diarrhea virus (BVDV) and classical swine fever virus (CSFV), similarly are dependent on the cellular miR-17 family, although binding occurs on the viral 3’UTR [16].

The unique GB virus B (GBV-B) isolate was the only known HCV-related hepacivirus until 2011. Using replicon systems, GBV-B appears to be only partially dependent on miR-122 [17]. However, recent discoveries have identified a plethora of HCV-related viruses in horses, rodents, bats, monkeys, and cows [18–25], most if not all of which contain miR-122 seed sites in the 5’UTR. However, the miR-122 requirement has not been investigated for these viruses, as no cell culture systems have been established [24,26].

The equine non-primate hepacivirus (NPHV) shares the highest sequence homology to HCV [18,27]. The genome structure of NPHV resembles HCV, with a long open reading frame (ORF) that can be translated into a 2942 amino acid long polypeptide. The polypeptide is predicted to be cleaved into the ten viral proteins C, E1, E2, p7, NS2, NS3, NS4A, NS4B, NS5A, and NS5B. An IRES structure similar to that for HCV was predicted for the NPHV 5’UTR, but with a much longer stem loop 1 (SL1) structure and only one predicted miR-122 binding site. Using NPHV IRES reporter systems, we previously showed a pro-translational role for miR-122 [28].

Here, we aimed to obtain a broader understanding of hepacivirus miRNA dependence and how this might influence tissue tropism. To understand miR-122 requirements for NPHV, we used AGO-CLIP *in vivo* to pinpoint the miRNA interactome on viral RNA, and developed viable NPHV/HCV 5’UTR chimeras. This approach suggested that NPHV is only partially dependent on miR-122. Encouraged by this result and to determine whether miRNA independent hepaciviruses are viable, we randomized the miRNA seed site of these chimeras and HCV in an attempt to develop miRNA independent viruses. Our results indicated that hepaciviruses have the potential to escape miRNA requirements and break the liver-specific tissue tropism barrier. It appears that such variants must have been sampled during hepacivirus evolution. Interestingly, miR-122 dependence still has been strongly selected, possibly to leverage the tolerogenic liver environment to establish and maintain chronic infection.

## Results

### NPHV interacts with miRNAs at conserved miR-122 sites *in vivo*

**Fig 1:**
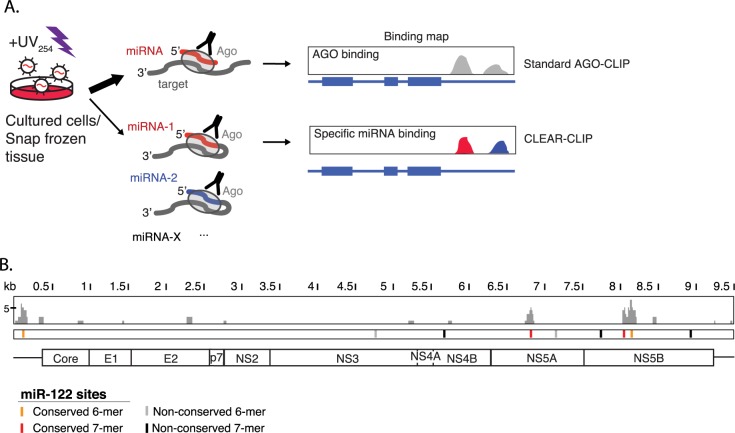
AGO-CLIP provides a miRNA binding map for NPHV *in vivo*. **(A)** Schematic of standard AGO-CLIP and CLEAR-CLIP. After UV-induced cross-linking of RNA-protein complexes, precipitation of the AGO complex and RNA library preparation, standard AGO-CLIP provides a map of AGO/miRNA interactions across the transcriptome. In CLEAR-CLIP, chimeras of miRNAs with cellular or viral RNAs are induced. Analysis of these allows unambiguous detection of specific miRNA interactions. **(B)** *In vivo* AGO/miRNA binding across the NPHV genome from horse liver. Binding is observed across the four miR-122 seed sites conserved among all published isolates. Non-conserved sites present in the NZP1 isolate are indicated.

To investigate putative NPHV/miR-122 interactions, we performed AGO-CLIP on liver biopsies from two NPHV infected horses. Although sequence coverage on viral RNA was much lower compared to highly infected HCV cell cultures [15] negating our ability to unambiguously identify interacting miRNAs [16] (Fig 1A), this assay identified regions with AGO/miRNA interactions across the NPHV genome. Interestingly, all significant peaks perfectly overlapped the four conserved miR-122 sites; one in the 5’UTR, one in NS5A and two in NS5B (Fig 1B). These data strongly suggest interaction with miR-122 during NPHV infection *in vivo*.

### NPHV/HCV chimeras containing regions of the NPHV 5’UTR can establish replication and virus production in Huh-7.5 cells

**Fig 2:**
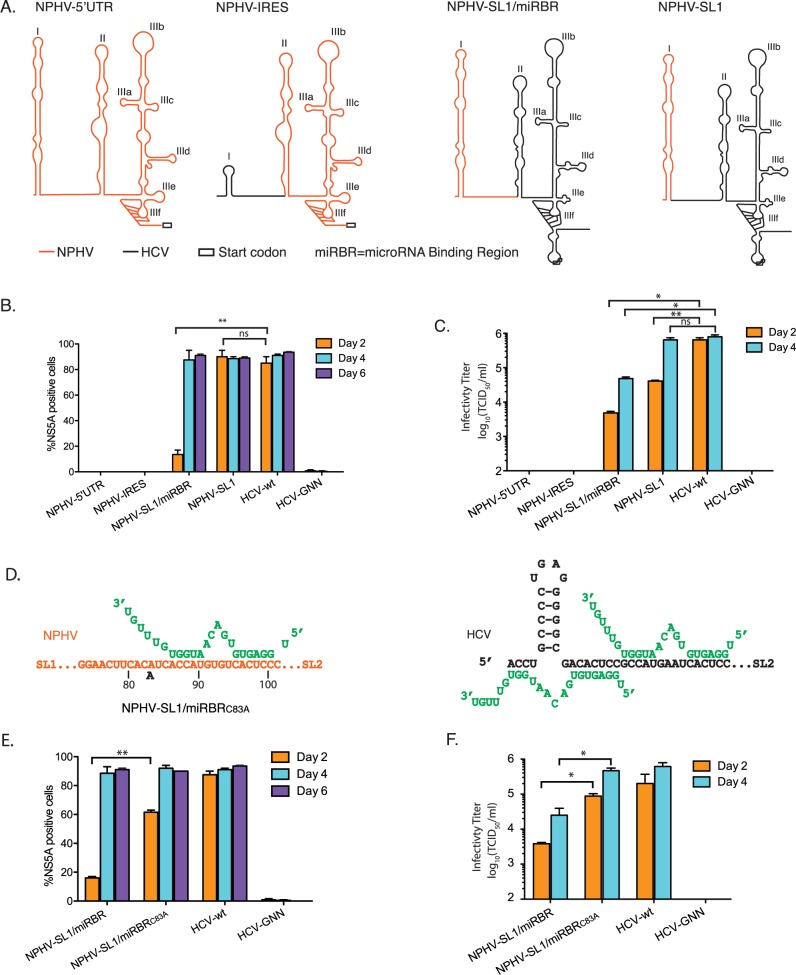
Identification of NPHV/HCV chimeras capable of replication and infectious virus production in Huh-7.5 cells. **(A)** Schematic showing the secondary structures of 5’UTR regions of HCV/NPHV chimeras that contain the NPHV entire 5’UTR (NPHV-5’UTR), IRES (NPHV-IRES), Stem Loop 1 with microRNA Binding Region (miRBR) (NPHV-SL1/miRBR), or only SL1 (NPHV-SL1). All chimeras were constructed on the HCV J6/JFH Clone 2 backbone. **(B,E)** NS5A positive cells post transfection of Huh-7.5 cells. Results represent mean±SEM from 3 independent transfections. **(C,F)** Infectious virus production quantified by limiting dilution assay on naïve Huh-7.5 cells post transfection (n = 3). **(D)** Schematic of predicted miR-122 binding modes to NPHV and HCV. The adaptive mutation of NPHV-SL1/miRBR at C83A site is indicated. Asterisks, *p < 0.05, **p < 0.01, Student’s t test.

To functionally characterize NPHV miR-122 requirements in the absence of a cell culture system supporting NPHV replication, we set out to establish NPHV/HCV chimeras based on the HCV genotype 2a recombinant J6/JFH Clone2 [29]. We constructed four different NPHV/HCV chimeras to test regions of the HCV 5’UTR that could be replaced by NPHV (Fig 2A). Following transfection of these chimeric genomes into Huh-7.5 cells, we assayed viral replication by staining for NS5A positive cells and measured virus production by limiting dilution (TCID_50_; Fig 2B and 2C). Replacement of the entire 5’UTR or IRES region (NPHV-5’UTR and NPHV-IRES) abolished replication completely, even when followed for 29 days. In contrast, NPHV-SL1 infection spread similarly to the parental HCV construct, although virus production was slightly delayed (Fig 2B and 2C). This indicated that exchange of SL1 had only limited effect on replication efficiency. Although NPHV-SL1/miRBR (microRNA Binding Region) was attenuated, it spread to the majority of cells on day 4 with virus production ~10-fold lower than the parental HCV recombinant (Fig 2B and 2C). Since NPHV-SL1/miRBR contains the miR-122 binding site of NPHV, this chimera was of particular interest for studying NPHV miR-122 dependence. We tested the IRES activity of NPHV-5’UTR and NPHV-IRES to distinguish whether the absence of NS5A positive cells was due to block of replication or translation. Compared to HCV, the luciferase signal driven by the NPHV-5’UTR and NPHV-IRES variants was about 3-fold lower, but still much higher than the background (S1 Fig). This suggests that the failure of these viruses to replicate is not due to a block in translation.

### A point mutation in the single stranded region of NPHV-SL1/miRBR increases both replication efficiency and virus production

To determine whether the NPHV-SL1/miRBR recombinant could be further adapted, we took supernatant on day 6 after transfection and infected naïve Huh-7.5 cells. Supernatant from newly infected cells was then harvested on day 6 and the 5’ end of the viral genome was sequenced. This analysis revealed a C83A mutation in NPHV-SL1/miRBR upstream of the miR-122 site (including putative auxiliary pairing) (Fig 2D). This change did not facilitate binding of a second miR-122 molecule; rather, it changed this region further from mirroring the HCV seed site 1. To confirm the impact of the C83A mutation, we introduced this nucleotide change into the original NPHV-SL1/miRBR genome and again transfected Huh-7.5 cells. The mutant exhibited superior replication and virus production compared to the original NPHV-SL1/miRBR, and was now only slightly attenuated compared to the HCV parent (J6/JFH1-Clone2), as judged by spread of infection and virus yield (Fig 2E and 2F).

### miR-122 is only partially required for replication and virus production of NPHV/HCV chimeras

**Fig 3:**
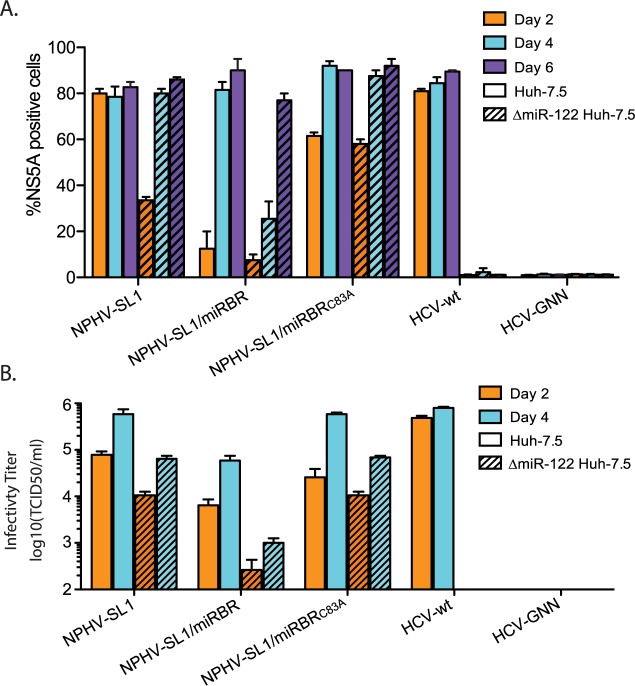
Growth kinetics of wild type HCV and NPHV/HCV chimeras in ΔmiR-122 Huh-7.5 cells. **(A)** NS5A positive cells post transfection in Huh-7.5 (open bars) and ΔmiR-122 Huh-7.5 cells (hatched bars). Results represent mean±SEM from 3 independent transfections. **(B)** Infectious virus production of supernatants from transfected ΔmiR-122 Huh-7.5 and Huh-7.5 cells. Virus titers were quantified by limiting dilution assay on naïve Huh-7.5 cells (n = 3).

Using a CRISPR engineered miR-122 knockout (KO) cell line, we next examined the ability of NPHV-SL1/miRBR to replicate in the complete absence of miR-122. As shown before, HCV replication was dramatically impaired in the miR-122 KO cell line (Fig 3A and 3B; [15]). The number of NS5A positive cells and viral infectivity titers of NPHV-SL1, NPHV-SL1/miRBR and NPHV-SL1/miRBR_C83A_ were also reduced. Nonetheless, replication and virus production for the NPHV/HCV chimeras was evident in cells lacking miR-122 (Fig 3A and 3B). These results indicated that the loss of miR-122 decreased NPHV replication efficiency, but that replication could still occur in complete absence of miR-122.

### miR-122 is the sole miRNA that binds to the miRBR in the NPHV 5’UTR

**Fig 4:**
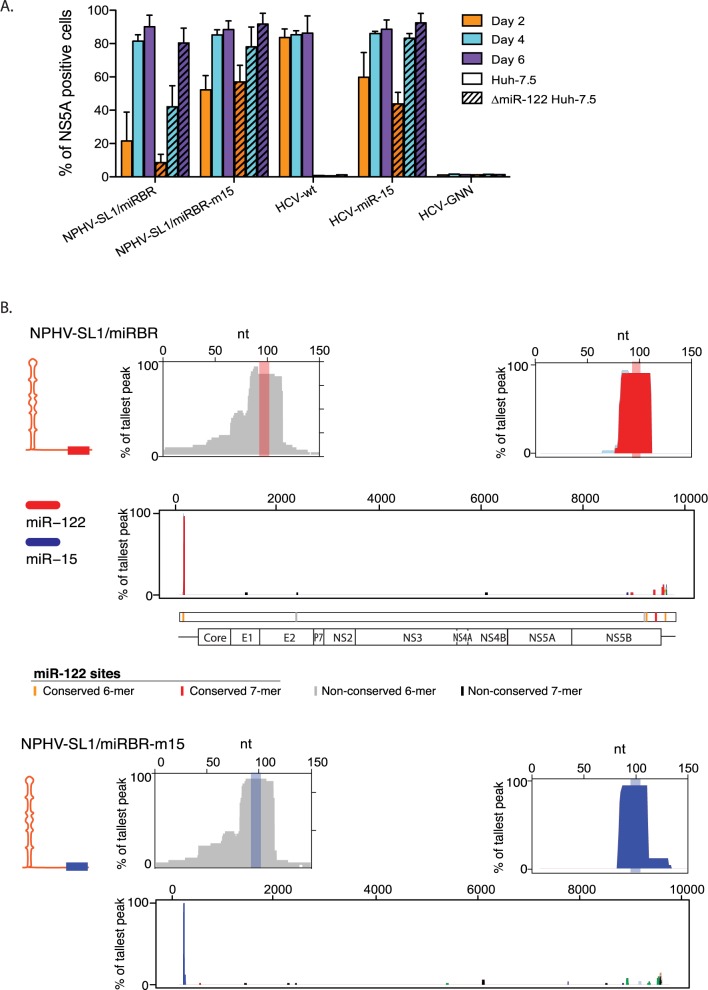
miR-122 but not other miRNAs binds the NPHV 5’UTR. **(A)** Percentage of NS5A positive ΔmiR-122 Huh-7.5 or Huh-7.5 cells post-transfection with the indicated virus constructs. Results represent mean±SEM from 3 independent transfections. **(B)** Standard AGO (left) and miRNA-specific chimera-derived (right) binding maps on the 5’ end of NPHV-SL1/miRBR (top) and NPHV-SL1/miRBR-m15 (bottom) in Huh-7.5 cells four days post-transfection. Chimera-supported specific miRNA binding across the entire viral genome is shown below for both panels. The binding site and chimera-supported interactions for miR-122 are shown in red and those of miR-15 in blue.

Next, we asked whether miRNAs other than miR-122 bind the NPHV miRBR. To this end, we first replaced the miR-122 binding site of NPHV-SL1/miRBR with a miR-15/16-binding sequence. Similar to an HCV variant with both miR-122 sites replaced by miR-15 sites [15], this virus was viable (Fig 4A). As expected, the HCV and NPHV-SL1/miRBR miR-15 variants were unaffected by the absence of miR-122 (Fig 4A). We then used AGO-CLIP and miRNA-target chimeras [16,30] to unambiguously identify interacting miRNAs in wt Huh-7.5 cells. For NPHV-SL1/miRBR and NPHV-SL1/miRBR-15, miR-122 and miR-15/16, respectively, were the only interacting miRNA species (Fig 4B). This confirmed that no other functional miR-122 site than the single canonical site exists in the NPHV miRBR, and that no other miRNA is binding this region.

### Isolation of miR-122 independent HCV and NPHV/HCV variants

**Fig 5:**
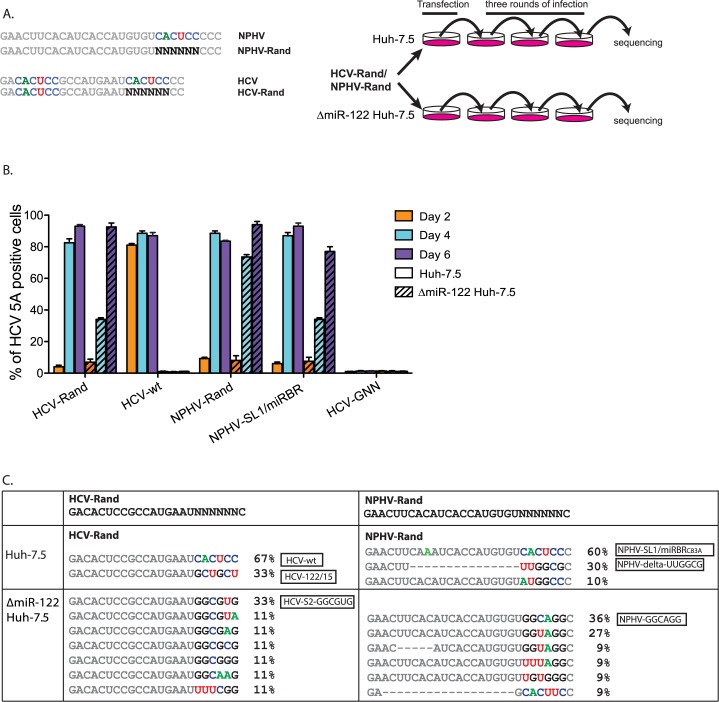
Evolutionary selection of seed site-randomized HCV and NPHV/HCV chimeras. **(A)** Schematic of the saturated mutagenesis approach. Left panel: comparison of NPHV-Rand and HCV-Rand sequence with the parental strains. miRNA binding sites are shown in color. Right panel: ΔmiR-122 Huh-7.5 cells and Huh-7.5 cells were transfected by NPHV-Rand or HCV-Rand, supernatants were collected and used to inoculate naïve cells of the same type. After three rounds of infection, the miRBR sites of enriched strains were sequenced. **(B)** Percentage of NS5A positive cells post-transfection in ΔmiR-122 Huh-7.5 and Huh-7.5 cells. Results represent mean±SEM from 3 independent transfections. **(C)** Summary of miRBR region sequences for NPHV-Rand and HCV-Rand enriched in ΔmiR-122 Huh-7.5 cells or Huh-7.5 cells. Residues of NPHV-SL1-miRBR and HCV corresponding to the miR-122 seed site are shown in color (C is shown in blue, A in green, G in black, and U in red). The isolates selected for follow-up reverse genetic studies are highlighted by boxed names.

Given the only partial NPHV miR-122 dependence, we next probed whether miRNA independent hepaciviruses with extrahepatic replication potential could be selected. We exploited the rapid evolutionary capacity of RNA viruses to select for variants with high fitness in the presence (Huh-7.5) or absence (ΔmiR-122 Huh-7.5) of miR-122. To increase input diversity, we randomized the miR-122 binding site of NPHV-SL1/miRBR to create NPHV-Rand. In parallel, we randomized the corresponding seed site 2 of HCV to create HCV-Rand (Fig 5A). To enrich for the most efficient variants, we took the supernatant of transfected cells when at least 50% of cells were NS5A positive to inoculate naïve cells (Fig 5A and 5B). After three passages, we sequenced the 5’UTR of the selected viruses in the supernatant. For HCV-Rand in Huh-7.5 cells, the majority (67%) contained the wildtype miR-122 binding site, whereas the rest (33%) contained the miR-15 binding site. Interestingly, the latter variant was identical to a synthetic miR-122/15 construct we previously showed to be viable [15]. In ΔmiR-122 Huh-7.5 cells, most recovered variants contained a G-rich region; among them 66% had the sequence GGCGNG. Similarly, most recovered NPHV-Rand variants from wild type cells contained the wild type miR-122 binding site. These had also acquired the previously described C83A mutation. Surprisingly, 30% of the recovered strains had a 15-nucleotide deletion in the miRBR region (NPHV-delta-UUGGCG). In ΔmiR-122 Huh-7.5 cells, 73% of the recovered NPHV variants had another G-rich GGYAGG motif. One variant from each group was selected for further characterization (Fig 5C, boxed names).

### Randomized, selected HCV and NPHV/HCV chimeras replicate in the absence of any miRNAs

**Fig 6:**
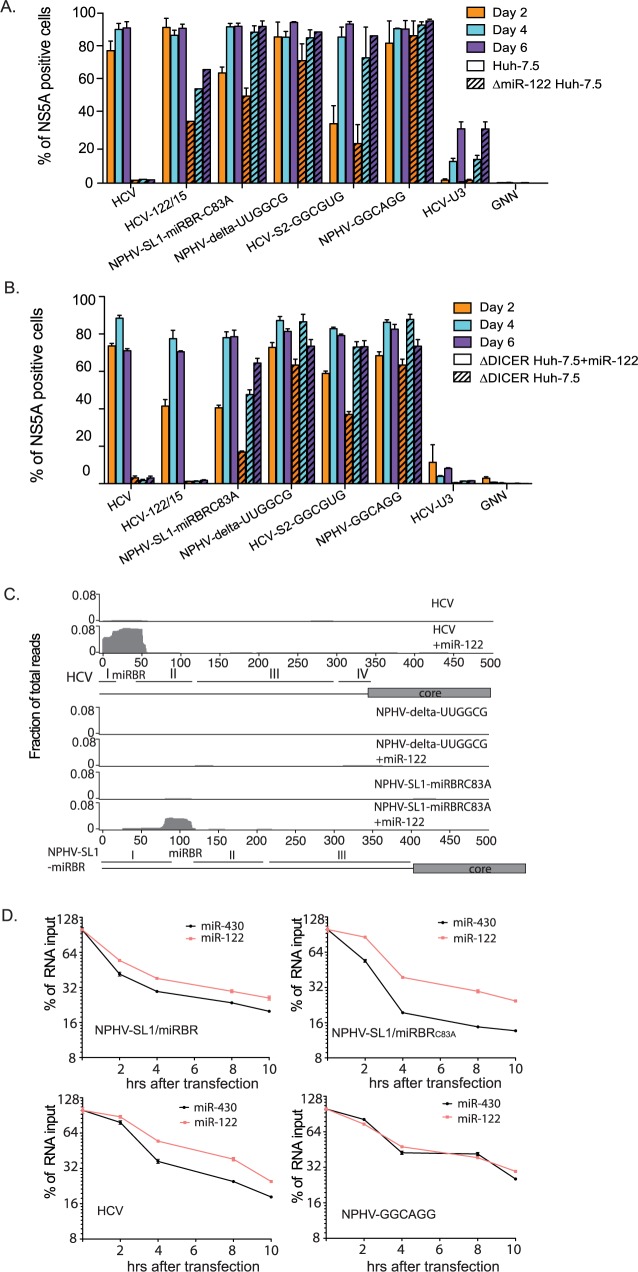
Evolutionary-selected NPHV/HCV variants replicate in the absence of any miRNA. **(A)** Percentage of NS5A positive ΔmiR-122 Huh-7.5 or Huh-7.5 cells post-transfection with the indicated viral constructs. Results represent mean±SEM from 3 independent transfections. **(B)** Percentage of NS5A positive ΔDICER Huh-7.5 cells post-transfection with the indicated viral constructs with (open bars) or without (hatched bars) co-transfection of miR-122. Results represent mean±SEM from 3 independent transfections. **(C)** AGO-CLIP binding maps for HCV (top), NPHV-delta-UUGGCG (middle) and NPHV-SL1/miRBR_C83A_(bottom) in ΔDICER Huh-7.5 cells 72hrs post-transfection with and without co-transfection of miR-122. Schematics of the 5’UTRs and initial core regions of HCV and NPHV are shown below. **(D)** Effect of miR-122 on HCV and NPHV/HCV RNA stability. RNA decay in ΔDICER Huh-7.5 cells was measured at the indicated times post-transfection in the presence of either miR-122 or miR-430 (50 μM each). RNA copy number at each time point was normalized to RNA copy number at 0hr (after transfection). The data is representative of 2 independent biological experiments.

We next engineered the selected sequences into the original viral genomes and tested their replicative fitness in Huh-7.5 and ΔmiR-122 Huh-7.5 cells. For comparison, we also included HCV-U3 in our analysis since this virus, which contains a fraction of the cellular U3 snoRNA sequence in place of the SL1 region, replicates in the absence of miR-122 [31]. Similar to HCV-U3, the selected variants HCV-S2-GGCGUG, NPHV-delta-UUGGCG and NPHV-GGCAGG all replicated comparably in the presence or absence of miR-122 (Fig 6A). The NPHV-based variants, however, were the most fit. HCV-122/15 replicated and spread comparably to parental HCV in Huh-7.5 cells, but was attenuated in the absence of miR-122. Thus, it appears that the selected NPHV-Rand variants in particular, could replicate with equal efficiently in the absence or presence of miR-122.

The sequences of the selected G-rich random variants did not correspond to known canonical miRNA seed sites. We therefore examined the ability of these variants to replicate in the complete absence of miRNAs. Using CRISPR mutagenesis we ablated DICER to produce ΔDICER Huh-7.5 cells. Given the critical role for DICER in cleaving pre-miRNAs [32,33], no mature miRNAs are produced in these cells. As expected, parental HCV and HCV-122/15 were not viable in ΔDICER Huh-7.5 cells (Fig 6B). Co-transfection of a synthetic miR-122 mimic (Fig 6B) rescued HCV and partially rescued HCV-122/15. In contrast, NPHV-SL1/miRBR_C83A_ and HCV-S2-GGCGUG replication was only slightly enhanced by miR-122 addition and NPHV-delta-UUGGCG and NPHV-GGCAGG spread with similar efficiency with or without miR-122.

To further confirm the absence of miRNA binding for these viruses, we performed AGO-CLIP of NPHV-delta-UUGGCG in ΔDICER Huh-7.5 cells compared to HCV and NPHV-SL1/miRBR_C83A_. As expected, replication and miRNA binding was observed for HCV only after addition of miR-122 (Fig 6C). The same result was found for NPHV-SL1/miRBR_C83A_ despite replication in miRNA deficient cells. No AGO/miRNA binding was observed for NPHV-delta-UUGGCG. These data prove that these selected NPHV/HCV variants can replicate in the complete absence of mature miRNAs. To determine whether miR-122 independent NPHV variants depend on miR-122 for RNA 5’ end protection, we measured the RNA stability of HCV, NPHV-SL1/miRBR, NPHV-SL1/miRBR_C83A_, and NPHV-GGCAGG in ΔmiR-122 Huh-7.5 cells. miR-122 but not miR-430 supplementation enhanced RNA stability, but only for miR-122 dependent variants (Fig 6D). Thus, although NPHV has a much larger SL1 structure, NPHV may still utilize miR-122 to enhance protection of the RNA from degradation. NPHV-GGCAGG, however, apparently does not need miRNA binding to protect its RNA against 5’ degradation.

### miR-122 independent NPHV/HCV variants but not those of HCV can replicate in extrahepatic cells

**Fig 7:**
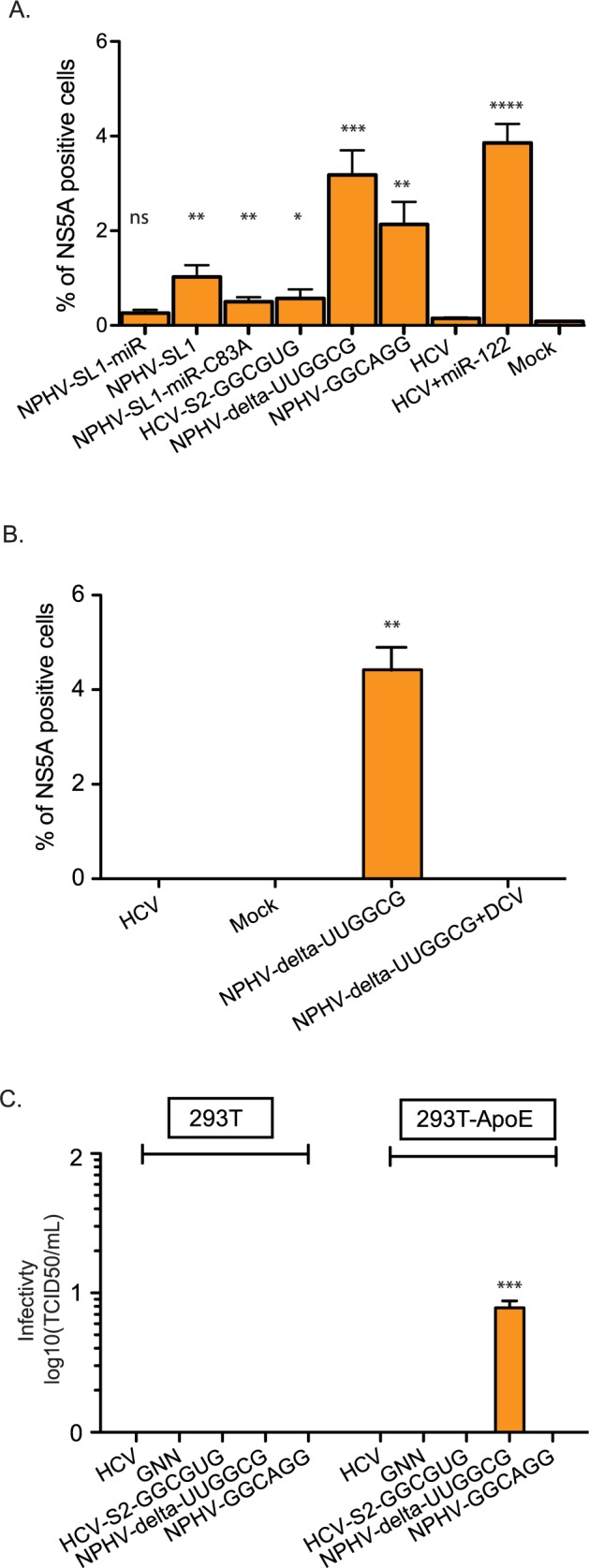
Evolutionary selected NPHV/HCV variants can replicate and produce infectious particles in non-hepatic cells. **(A)** Quantification of NS5A positive cells on day 2 post-transfection of 293T cells with HCV and NPHV/HCV variants. **(B)** Quantification of NS5A positive 293T cells post-transfection as in (A) but compared to a daclatasvir (DCV) treated culture. **(C)** Infectious virus production from 293T cells with or without exogenous ApoE expression. Virus titers were quantified by limiting dilution assay on naïve Huh-7.5 cells. In (A)-(C), results represent mean±SEM from 3–6 independent experiments. Asterisks, *p < 0.05, **p < 0.01, ***p < 0.001, ****p < 0.0001, Student’s t test.

miR-122 is expressed in Huh-7.5 cells [2], and reports showed that exogenous expression of miR-122 can facilitate efficient replication of HCV in other hepatic cell lines such as Hep3B and HepG2. Low-level replication was also observed in non-hepatic cells, including 293T kidney cells or engineered immortalized mouse fibroblasts (iMEF) [34–37]. We therefore tested whether the selected miR-122 independent strains could replicate in non-hepatic cells. As expected, HCV did not replicate in 293T cells (Fig 7A). In contrast, NS5A positive cells were observed for NPHV-SL1, NPHV-SL1/miRBR_C83A_ and HCV-S2-GGCGUG, although at very low frequencies. NPHV-delta-UUGGCG and NPHV-GGCAGG infected 2–5% of the cells similar to the frequency of HCV replication in these cells upon miR-122 addition. NPHV-delta-UUGGCG replication in 293T cells was completely abolished after addition of Daclatasvir, a potent NS5A inhibitor [38], thus confirming its authentic replication in 293T cells (Fig 7B).

ApoE is an essential factor for infectious HCV production, including in miR-122-supplemented 293T cells [39]. We therefore asked whether infectious particles could be produced in the presence of exogenous ApoE. In 293T cells transduced with a lentivirus expressing RFP-ApoE, low levels of infectious particles were produced by NPHV-delta-UUGGCG, but not by HCV, HCV-S2-GGCGUG or NPHV-GGCAGG, transfected cells (Fig 7C). No virus production was observed without ApoE expression. Despite the highly attenuated particle production in 293T-ApoE compared to Huh-7.5 cells, this proved that miRNA independent NPHV/HCV chimeras could replicate and produce infectious progeny in non-hepatic cells.

## Discussion

miR-122 has attracted great interest as a host requirement for HCV replication and hence a potential antiviral target. Blocking miR-122 leads to prolonged viral inhibition in cell culture, chimpanzees, and patients [5–7]. In addition, due to their long-lasting effects, miR-122 inhibitors are currently being considered for special patient populations where adherence to strict daily therapeutic regimens is problematic [40]. Therefore, it remains important to understand the miR-122 requirement of HCV and to study resistant variants that can replicate in miR-122 depleted environments. Furthermore, both the requirement of a host miRNA for an RNA virus, and the non-classical binding of miR-122 to the HCV 5’UTR are unique. We still have an incomplete understanding of how this interaction evolved and its biological importance.

NPHV is the closest homolog of HCV, making it an interesting comparative model. Understanding NPHV is also relevant given its possible association with equine liver disease [26,41]. Whereas all HCV genotypes have two conserved miR-122 binding sites, both being essential for replication [2,3], only one binding site was predicted for NPHV [18,19]. The length of the NPHV miRBR is comparable to that of HCV, raising the question whether a second miR-122 molecule binds in a non-canonical manner or, alternatively, a different miRNA binds this region. Our *in vivo* NPHV AGO-CLIP studies confirmed miRNA binding across the miR-122 site. We therefore took advantage of replication-competent NPHV/HCV chimeras engineered to contain parts of the NPHV 5’UTR. NPHV-SL1/miRBR was of particular interest, since it contains the NPHV miR-122 binding site. Although NPHV-SL1/miRBR had partially attenuated kinetics of viral protein accumulation and infectious virus production, more fit variants including NPHV-SL1/miRBR_C83A_, NPHV-delta-UUGGCG, and NPHV-GGCAGG could be readily selected in cell culture.

In our previous studies, we demonstrated that HCV miR-122 tropism could be replaced by other miRNAs, such as miR-15 [15]. It was therefore possible that the increased fitness of NPHV-SL1/miRBR_C83A_, NPHV-delta-UUGGCG, and NPHV-GGCAGG was acquired by binding to alternative miRNAs. However, we found that NPHV-SL1/miRBR_C83A_ bound only one molecule of miR-122, and neither NPHV-delta-UUGGCG nor NPHV-GGCAGG bound any miRNA. This was based on two observations: First, in contrast to HCV-m15, which could not replicate in ΔDrosha Huh-7.5 cells supplemented with miR-122 [15], NPHV-SL1/miRBR_C83A_ was not attenuated in ΔDICER Huh-7.5 cells supplemented with miR-122 compared to wt Huh-7.5 cells. The NPHV-delta-UUGGCG and NPHV-GGCAGG variants were not attenuated in ΔDICER Huh-7.5 cells or in ΔmiR-122 Huh-7.5 cells (Fig 6A and 6B). Second, miR-122 and miR-15, respectively, were the only miRNAs identified from AGO-CLIP chimeras at the miRBR region of NPHV-SL1/miRBR_C83A_ and NPHV-SL1/miRBR-m15. This was in contrast to the identification of two independent peaks for miR-15 and miR-122 for HCV122/15 and HCV15/122 [16]. Furthermore, no miRNA peak could be observed for NPHV-delta-UUGGCG, even after addition of miR-122 (Fig 6C). Taken together these results strongly suggest that newly adapted NPHV/HCV variants do not bind any other miRNA. The cell culture selected HCV-U3 variant, which is resistant to miR-122 inhibition [31], contains a large extended SL1 and only one miR-122 site. This 5’UTR is therefore structurally similar to NPHV. A larger 5’-terminal SL1 may at least partially compensate for miR-122 binding, possibly by preventing degradation by exonucleases. This suggests that it is possible to replace one miR-122 seed site and obviate miR-122 dependence (at least in part) by combining a larger SL1 with sequence changes in the miRBR region.

It is interesting to speculate why HCV requires two molecules of miR-122, whereas related hepaciviruses such as NPHV, require only one. From the current and previous studies, it is evident that HCV replication is attenuated if only one copy of miR-122 is bound. This includes mutants of individual seed sites [2,4,11,15,42] and the current study of HCV-S2-GGCGUG in Huh-7.5 cells and HCV-122/15 in ΔmiR-122 Huh-7.5 cells. Interestingly, the C83A mutation of NPHV-SL1/miRBR may induce a conformational change in the miRBR region to a more relaxed structure (S2A and S2B Fig). Although it is tempting to speculate that this more relaxed RNA structure might facilitate miR-122 access to the miRBR region, e.g. by providing access to auxiliary pairing with the ACC motif at nt 87–89, the C83A mutation also facilitated more efficient infection kinetics in the absence of miR-122 (Fig 3A and 3B), indicating that mechanisms beyond miR-122 engagement may be at play. HCV, HCV_G28A_ from a previous study [42], NHPV-delta-UUGGCG, (S1D–S1F Fig), but not NPHV-GGCAGGG or HCV-S2-GGCGUG (S1C and S1G Fig) have similar relaxed structures, suggesting that these predicted secondary structures of miRBR do not necessarily correlate with miR-122 dependency. We identified NPHV-SL1/miRBR variants that are either partially or completely independent of miR-122 yet we could not identify any completely independent HCV-based variants, presumably because seed site 1 of HCV was still intact (Fig 6A). Interestingly, NPHV-delta-UUGGCG, but not HCV variants, could produce infectious viruses in 293T kidney cells expressing ApoE (Fig 7C). Since miR-122 is liver specific and remains an important factor for HCV tropism, the fact that NPHV only requires one molecule of miR-122 could lower the threshold for NPHV to infect other organs. However, except for sporadic evidence of extrahepatic presence of NPHV [18,41], it remains to be determined if replication occurs in other tissues *in vivo*. Still it remains feasible that low-level replication outside the liver could occur and influence the course of disease or transmission between hosts.

It was striking, but not surprising, that the fittest HCV variant emerging from our saturation mutagenesis library in wild type Huh-7.5 cells carried the wild type seed sites. This confirms that in the Huh-7.5 environment miR-122 binding HCV is indeed the optimal variant (Fig 5C). Another interesting finding was that HCV-122/15, which we previously predicted and confirmed to be viable, was also selected in Huh-7.5 cells. HCV therefore appears to function most efficiently with two miRNA seed sites. While miR-122 could be substituted by other miRNAs, HCV with two miR-122 sites remains superior. In contrast, NPHV either acquired the wild type miR-122 site in combination with an adaptive mutation (NPHV-SL1/miRBR_C83A_) or carried a 15-nt truncation (NPHV-delta-UUGGCG) in the miRBR. Thus, miR-122 binding NPHV variants were also selected as long as miR-122 was available.

We were able to isolate NPHV and HCV variants in the ΔmiR-122 Huh-7.5 cells with similar efficiency (Fig 6A). This makes it likely that during hepacivirus evolution miR-122-indepedent variants have emerged. It is therefore curious why most hepaciviruses isolated thus far retain at least one miR-122 binding site and hence hepatocyte tropism. Clearly, basic replicative functions can ensue in the absence of miR-122 using alternative 5’UTR structures and/or binding sites for different miRNAs. We therefore speculate that sampling of hepaciviruses in nature has been biased to those that can establish chronic infection in their animal hosts. Retaining miR-122 dependence and restricting replication to hepatocytes may allow these viruses to take advantage of the tolerogenic liver environment [43,44] to establish and maintain chronicity. Variants with the ability to replicate in other cell types might be selected against if they elicit an adaptive immune response that eliminates infection and prevents chronicity. Consequently, we posit that there may be hepaciviruses in nature that are not strictly hepatotropic and that these viruses will be more likely to cause acute resolving infections with different modes of transmission to ensure their survival.

Besides the interaction of miR-122 with the 5’UTR of NPHV, our *in vivo* AGO-CLIP studies demonstrated miRNA binding at all three other conserved miR-122 sites in the NPHV polyprotein coding region (Fig 1B). Unfortunately, we lack the cell culture systems needed to assess the potential contributions of miRNA binding at these NS5A/5B sites. Thus, it remains possible that these sites also contribute towards NPHV miR-122 dependence. For HCV, however, miR-122 sites in the ORF and 3’UTR do not appear to influence viral replication and/or production [15,45]. It will be interesting to explore this further using infectious clones *in vivo*, or after the development of tractable NPHV culture systems [28].

In conclusion, using a panel of NPHV/HCV chimeras, we were able to test the miRNA dependence of NPHV in cell culture. We demonstrate that one molecule of miR-122 is the only miRNA that binds the NPHV miRBR region, consistent with our *in vivo* data. Using NPHV/HCV chimeras, miR-122 independent variants could be selected that were capable of extra-hepatic replication. This indicates that the interaction of one molecule of miR-122 with the NPHV miRBR does contribute to its hepatic tropism but that subtle changes in the NPHV 5’UTR have higher potential to break the tissue tropism barrier as compared to HCV. Given that most hepaciviruses observed in nature contain at least one miR-122 binding site despite the fact that minor changes in the miRBR can weaken or even obviate miR-122 dependence indicates a strong selective pressure to maintain hepatotropism. We suggest that this selective force is not for basic hepacivirus replicative functions but rather to exploit the tolerogenic liver environment to orchestrate chronicity.

## Materials and methods

### Ethics statement

We used NPHV infected liver biopsies from two horses. This did not require euthanasia of any animal. Ultrasound-guided percutaneous biopsies were taken from horses, using standard procedures at the College of Veterinary Medicine, Cornell University and adhered to the Institutional Animal Care and Use Committee protocol at this institution.

### Construction of NPHV plasmids

NPHV-5’UTR, NPHV-IRES, NPHV-SL1/miRBR, and NPHV-SL1 were constructed in the HCV J6/JFH Clone2 backbone [29], by replacing the 5’UTR (nt 1–341), IRES (43–341), SL1-miRBR (1–42), or SL1 (1–20) of HCV by the corresponding sequence of the 5’UTR (1–384), IRES(103–384), SL1-miRBR (1–102) or SL1(1–74) of the NZP1 NPHV isolate [28]. HCV-122/15 and HCV-S2-GGCGUG were constructed by replacing seed site 2 of HCV (37–42) with the miR-15 binding site (GCTGCT) or GGCGTG, respectively. SL1-miRBR_C83A_, NPHV-GGCAGG, and NPHV-delta-UUGGCG were constructed from NPHV-SL1/miRBR by introducing single mutations or exchanging the miRBR site (also see Fig 5C). NPHV-SL1/miRBR-m15 was constructed by replacing the miR-122 binding site at position of 96–101 of NPHV-SL1/miRBR with the miR-15 binding site (GCTGCT). For TRIP-hApoE3shres-TagRPF, the cDNA clone of human apolipoprotein E (NM_001302691) was amplified from Huh-7.5 cDNA using the primers RU-O-19451 and RU-O-19452 that contains MluI and BamHI sites. This was then cloned into pDONOR221 (Fisher Scientific), which was linearized by the same pair of endonucleases. All primer sequences are listed in S1 Table.

### Cell culture

Huh-7.5 [46] and ΔmiR-122 Huh-7.5 hepatoma cells [15], derived previously in our laboratory, were maintained in Dulbecco's Modified Eagle Medium (DMEM, Invitrogen) supplemented with 0.1 mM nonessential amino acids (Invitrogen) and 5% fetal bovine serum (FBS). 293T cells (ATCC) were maintained in DMEM supplemented with 10% FBS and 0.1 mM NEAA as described [47]. ΔDICER Huh-7.5 hepatoma cells were generated as described below and maintained in DMEM supplemented with 10% FBS and 0.1 mM NEAA. To produce 293T-ApoE, 293T cells were transduced with lentiviruses produced from pTRIP-hApoE3shres-TagRFP in a 293T producer culture, as previously described [37]. Efficiency of transduction was confirmed by the percentage of RFP positive cells before further analysis. Three different 293T cell lines derived from single colonies of 293T cells were tested to confirm the consistent observation of NS5A positive cells with NPHV-delta-UUGGCG. For Daclatasvir (DCV) treatment, 293T cells were pre-incubated with 10 nM of DCV (100-fold EC_50_[48]) one day before transfection with viral RNA.

### CRISPR mediated deletion of DICER in Huh-7.5 cells

To make ΔDICER Huh-7.5 cells, we deleted exons 2 and 19 in the DICER gene in Huh-7.5 cells described above. Guide sequence pairings were as follows: Dicer.sgRNAEX2.1a with Dicer.sgRNAEX2.1b, Dicer.sgRNAEX2.2a with Dicer.sgRNAEX2.2b, Dicer.sgRNAEX19.1a with Dicer.sgRNAEX19.1b, and Dicer.sgRNAEX19.2a with Dicer.sgRNAEX19.2b. Guide RNAs were cloned into pX458-SpCas9-(BB)-2A-GFP (Addgene, #48138). After sequence confirmation, transfection and single cell dilution, cloning proceeded as previously described [15]. To genotype single cell clones and to approximate editing efficiency in bulk cells, DNA was extracted using QuickExtract (Epicenter) and DICER exon 2 and 19 loci were PCR amplified using primers DicerEX2_GenomicF with DicerEX2_GenomicR, and DicerEX19_GenomicF with DicerEX19_GenomicR, respectively. The resulting PCR products underwent gel electrophoresis. The sole surviving homozygous deletion clone was expanded.

### *In vitro* transcription and RNA transfection

NPHV/HCV and HCV recombinant RNAs were *in vitro* transcribed from *XbaI* linearized DNA plasmids using T7 RiboMAX Express Large Scale RNA Production System (Promega). RNA was treated with RQ1 DNase (Promega) at 37°C for 15 min and purified on RNeasy columns (Qiagen).

For transfection of Huh-7.5 based cell lines, 1 μg RNA was mixed with 5 μL Lipofectamine 2000 (Life Technologies) in 500 μL OptiMEM, incubated 10 min and added to 3.5x10^5^ cells in 6-well plates where media was changed to DMEM containing 1.5% FBS and 1% NEAA before transfection. For transfection of 293T-based cell lines, transfection was performed with 5x10^5^ cells. 10 pmol of miR-122 was co-transfected with viral RNAs where indicated. To increase the transfection efficiency, cells were then spinoculated for 30 min at 37°C with 1000 g. Cells were split every 2 days, 1/3 of cells were seeded for the next time point, 2/3 of cells were pelleted, fixed with 2% PFA, and used for flow cytometry detection of HCV NS5A expression by staining with the 9E10 antibody conjugated with Alexa 647. Supernatant aliquots were stored at -80°C for virus titration assays (TCID_50_). HCV infectious titers were determined by a limiting dilution assay on naïve Huh-7.5 cells as previously described [49].

### Saturated mutagenesis assay

To generate saturated libraries of NPHV-Rand and HCV-Rand containing all possible combinations of nucleotides in the randomized region, we constructed bacterial transformation libraries with at least 10 times more colonies than the combination of all possible nucleotides (4^6 = 4096). Briefly, we introduced randomized seed sites into NPHV-SL1/miRBR and HCV by PCR with RU-O-21135 and RU-O-17131 or RU-O-21134 and RU-O-17131, respectively. After restriction digest with *Not*I and *Kpn*I, the PCR product was ligated into the parental plasmid and transformed into DH10B with electroporation transformation. After transformation, cells were revived in 500 μl SOC medium. 2 μl were spread on P10 LB medium to count colonies. To minimize potential bias of bacterial growth, bacteria were spread onto 2xP500 LB plates. After overnight incubation, cells were scraped into 20 ml LB medium and plasmids were purified with Plasmid Maxi-prep kit (Qiagen) without further expansion.

### Sequencing of viral RNA from supernatants

For selected viruses, as indicated in the main text and figures, the 5’UTRs of viruses present in the supernatant was sequenced using 5’RACE according to manufacturers protocol (Invitrogen). The primer RU-O-17102 was used for reverse transcription and RU-17104 and RU-18654 for the subsequent 1^st^ and 2^nd^ PCRs, respectively. PCR products were then cloned into TOPO-TA vectors (Invitrogen) for sequencing. Corresponding plasmids were included in the same procedure as negative control.

### CLIP assay

Standard AGO-CLIP was done as described [50]. To enable cost-effective multiplexing, we added sequencing adapters and 5’ indices in the 2^nd^ PCR step using the primers listed in S1 Table. This strategy uses the DP5 and DP3 sequences of the 1^st^ PCR product as priming sites to add 5’ indices and 3’ adapters for a short (6–16 cycles) 2^nd^ PCR step.

CLEAR-CLIP is based on standard AGO-CLIP with modifications to enrich for miRNA-target chimeras [30]. Briefly, the following procedures replace the post-immunoprecipitation steps of standard AGO-CLIP: (i) 5’-end phosphorylation using PNK (3’ phosphatase minus), (ii) Over-night chimera ligation using T4 RNA ligase 1, (iii) Alkaline phosphatase treatment to remove 3’ phosphate groups, (iv) 3’-linker ligation using truncated T4 RNA ligase 2 and pre-adenylated linker (using a pre-adenylated linker and omitting the enzyme in step ii allows negative controls to distinguish cellular vs. on-bead ligation events), and (v) Radio labeling using T4 PNK and [γ-^32^P]-ATP. Here, the ligase-free controls used to establish the method [30] were not needed, and the 3’ linker ligation was therefore done with T4 RNA ligase 1 and a radioactively labeled phosphorylated RNA linker according to the standard AGO-CLIP protocol [50], and not using truncated T4 RNA ligase 2 and a pre-adenylated linker. Accordingly, the subsequent PNK treatment was done with non-radioactive ATP.

For *in vivo* experiments, NPHV infected liver biopsies from two horses were taken and snap-frozen at the College of Veterinary Medicine, Cornell University adhering to Institutional Animal Care and Use Committee protocols as previously described [28]. Samples were powderized in liquid nitrogen followed by cross-linking with UV 254nm 3x at 400mJ/cm^2^. Results were combined from a 54mg biopsy from day 23 of an acutely infected horse (by intrahepatic RNA inoculation, NZP1 strain) with a serum titer of 10^6.7^ GE/mL and a 137mg biopsy of a chronically NPHV infected horse with a serum titer of 10^5^ GE/mL. Cultured Huh-7.5 cells for CLIP were transfected with NPHV-SL1/miRBR or NPHV-SL1/miRB-m15 and cross-linked 4 days post transfection; HCV, NPHV-SL1/miRBR_C83A_, HCV-S2-GGCGUG, and NPHV-delta-UUGGCG, either in the absence or presence of miR-122 in ΔDICER Huh-7.5 cells were cross-linked 3 days post transfection.

### RNA degradation assay

1 `μg of HCV-p7ns2Gluc-GNN or NPHV-p7ns2Gluc-GNN RNA, synthesized as previously described [51], was co-transfected in the presence of miR-122 or miR-430 mimic (Dharmacon) using the transfection protocol described above for ΔDICER Huh-7.5 cells in 6-well plates. At each time point, cells were washed with PBS 5 times and detached using trypsin. Cells were pelleted at 500 g, 4°C for 5 min and washed once with 1 mL of cold PBS. Cells were then pelleted under the same conditions and resuspended in 200 μL PBS. Cells were stored at -80°C or processed immediately by adding 1 mL Trizol. Samples were then mixed and 200 μL of chloroform were added and processed as described. After phase separation, the upper phase (approximately 600 μL) was transferred to another RNase free tube, mixed 1:1 with ethanol (100%) and loaded on RNeasy mini kit column (Qiagen). RNA was further purified as described and quantified by a two-step procedure using the QuantiTect Reverse Transcription kit (Qiagen) for cDNA synthesis followed by a qPCR using SYBR Green PCR Master Mix (Applied Biosystems) at 95°C for 10 min followed by 40 cycles of 95°C for 15 sec, 60°C for 15 sec and 72°C for 15 sec using primers RU-O-17104 and RU-O-21903. Standard curves were generated from the same RNAs.

### Luciferase reporter assays

RNA transfection was done as described above using NPHV-p7ns2Gluc-GNN and the indicated derivative RNAs. At 6hr post transfection, the supernatant of each well was collected to assay for *Renilla* luciferase as described [15] and read on a FLUOstar Omega (BMG Labtech).

## Supporting information

S1 Fig IRES activity of HCV and NPHV variants. Huh-7.5 cells were transfected with replication-deficient HCV-p7ns2Gluc-GNN and NPHV-p7ns2Gluc-GNN variant RNAs, as indicated. At 6hr post transfection, the supernatant of each well was collected to assay for *Renilla* activity. All values were normalized to the HCV control. Data represent the mean (±sem) of three independent experiments.
(EPS)

S2 Fig Comparison of predicted secondary structures of miRBR region within HCV and NPHV constructs. The nucleotides corresponding to stem loop 1 (SL1) and microRNA binding region (miRBR) of NPHV **(A-D)** and HCV **(E-G)** are shown and folded using Mfold [52] (available at http://mfold.rna.albany.edu/?q=mfold). Mutagenized sites **(A,B,E,F)** and altered microRNA-122 binding sites **(C,D,G)** are boxed.
(EPS)

S1 Table Sequence of oligonucleotides. (XLSX)

## References

[1] GLOBAL HEPATITIS REPORT, 2017 (n.d.). Available: http://www.who.int/hepatitis/publications/global-hepatitis-report2017/en/.

[2] Jopling CL, Yi M, Lancaster AM, Lemon SM, Sarnow P (2005) Modulation of hepatitis C virus RNA abundance by a liver-specific MicroRNA. Science 309: 1577–1581. doi: 10.1126/science.1113329 16141076 10.1126/science.1113329

[3] Jopling CL, Schütz S, Sarnow P (2008) Position-dependent function for a tandem microRNA miR-122-binding site located in the hepatitis C virus RNA genome. Cell Host Microbe 4: 77–85. doi: 10.1016/j.chom.2008.05.013 18621012 10.1016/j.chom.2008.05.013 PMC3519368

[4] Machlin ES, Sarnow P, Sagan SM (2011) Masking the 5’ terminal nucleotides of the hepatitis C virus genome by an unconventional microRNA-target RNA complex. Proc Natl Acad Sci U S A 108: 3193–3198. doi: 10.1073/pnas.1012464108 21220300 10.1073/pnas.1012464108 PMC3044371

[5] Elmén J, Lindow M, Schütz S, Lawrence M, Petri A, et al (2008) LNA-mediated microRNA silencing in non-human primates. Nature 452: 896–899. doi: 10.1038/nature06783 18368051 10.1038/nature06783

[6] Lanford RE, Hildebrandt-Eriksen ES, Petri A, Persson R, Lindow M, et al (2010) Therapeutic silencing of microRNA-122 in primates with chronic hepatitis C virus infection. Science 327: 198–201. doi: 10.1126/science.1178178 19965718 10.1126/science.1178178 PMC3436126

[7] Janssen HL, Reesink HW, Lawitz EJ, Zeuzem S, Rodriguez-Torres M, et al (2013) Treatment of HCV infection by targeting microRNA. N Engl J Med 368: 1685–1694. doi: 10.1056/NEJMoa1209026 23534542 10.1056/NEJMoa1209026

[8] Li Y, Masaki T, Yamane D, McGivern DR, Lemon SM (2013) Competing and noncompeting activities of miR-122 and the 5’ exonuclease Xrn1 in regulation of hepatitis C virus replication. Proc Natl Acad Sci U S A 110: 1881–1886. doi: 10.1073/pnas.1213515110 23248316 10.1073/pnas.1213515110 PMC3562843

[9] Sedano CD, Sarnow P (2014) Hepatitis C virus subverts liver-specific miR-122 to protect the viral genome from exoribonuclease Xrn2. Cell Host Microbe 16: 257–264. doi: 10.1016/j.chom.2014.07.006 25121753 10.1016/j.chom.2014.07.006 PMC4227615

[10] Li Y, Yamane D, Lemon SM (2015) Dissecting the roles of the 5’ exoribonucleases Xrn1 and Xrn2 in restricting hepatitis C virus replication. J Virol 89: 4857–4865. doi: 10.1128/JVI.03692-14 25673723 10.1128/JVI.03692-14 PMC4403451

[11] Henke JI, Goergen D, Zheng J, Song Y, Schüttler CG, et al (2008) microRNA-122 stimulates translation of hepatitis C virus RNA. EMBO J 27: 3300–3310. doi: 10.1038/emboj.2008.244 19020517 10.1038/emboj.2008.244 PMC2586803

[12] Díaz-Toledano R, Ariza-Mateos A, Birk A, Martínez-García B, Gómez J (2009) In vitro characterization of a miR-122-sensitive double-helical switch element in the 5’ region of hepatitis C virus RNA. Nucleic Acids Res 37: 5498–5510. doi: 10.1093/nar/gkp553 19578061 10.1093/nar/gkp553 PMC2760801

[13] García-Sacristán A, Moreno M, Ariza-Mateos A, López-Camacho E, Jáudenes RM, et al (2015) A magnesium-induced RNA conformational switch at the internal ribosome entry site of hepatitis C virus genome visualized by atomic force microscopy. Nucleic Acids Res 43: 565–580. doi: 10.1093/nar/gku1299 25510496 10.1093/nar/gku1299 PMC4288189

[14] Masaki T, Arend KC, Li Y, Yamane D, McGivern DR, et al (2015) miR-122 stimulates hepatitis C virus RNA synthesis by altering the balance of viral RNAs engaged in replication versus translation. Cell Host Microbe 17: 217–228. doi: 10.1016/j.chom.2014.12.014 25662750 10.1016/j.chom.2014.12.014 PMC4326553

[15] Luna JM, Scheel TK, Danino T, Shaw KS, Mele A, et al (2015) Hepatitis C virus RNA functionally sequesters miR-122. Cell 160: 1099–1110. doi: 10.1016/j.cell.2015.02.025 25768906 10.1016/j.cell.2015.02.025 PMC4386883

[16] Scheel TK, Luna JM, Liniger M, Nishiuchi E, Rozen-Gagnon K, et al (2016) A Broad RNA Virus Survey Reveals Both miRNA Dependence and Functional Sequestration. Cell Host Microbe 19: 409–423. doi: 10.1016/j.chom.2016.02.007 26962949 10.1016/j.chom.2016.02.007 PMC4826034

[17] Sagan SM, Sarnow P, Wilson JA (2013) Modulation of GB virus B RNA abundance by microRNA-122: dependence on and escape from microRNA-122 restriction. J Virol 87: 7338–7347. doi: 10.1128/JVI.00378-13 23616647 10.1128/JVI.00378-13 PMC3700295

[18] Kapoor A, Simmonds P, Gerold G, Qaisar N, Jain K, et al (2011) Characterization of a canine homolog of hepatitis C virus. Proc Natl Acad Sci U S A 108: 11608–11613. doi: 10.1073/pnas.1101794108 21610165 10.1073/pnas.1101794108 PMC3136326

[19] Burbelo PD, Dubovi EJ, Simmonds P, Medina JL, Henriquez JA, et al (2012) Serology-enabled discovery of genetically diverse hepaciviruses in a new host. J Virol 86: 6171–6178. doi: 10.1128/JVI.00250-12 22491452 10.1128/JVI.00250-12 PMC3372197

[20] Kapoor A, Simmonds P, Scheel TK, Hjelle B, Cullen JM, et al (2013) Identification of rodent homologs of hepatitis C virus and pegiviruses. MBio 4: e00216–13. doi: 10.1128/mBio.00216-13 23572554 10.1128/mBio.00216-13 PMC3622934

[21] Drexler JF, Geipel A, König A, Corman VM, van Riel D, et al (2013) Bats carry pathogenic hepadnaviruses antigenically related to hepatitis B virus and capable of infecting human hepatocytes. Proc Natl Acad Sci U S A 110: 16151–16156. doi: 10.1073/pnas.1308049110 24043818 10.1073/pnas.1308049110 PMC3791787

[22] Quan PL, Firth C, Conte JM, Williams SH, Zambrana-Torrelio CM, et al (2013) Bats are a major natural reservoir for hepaciviruses and pegiviruses. Proc Natl Acad Sci U S A 110: 8194–8199. doi: 10.1073/pnas.1303037110 23610427 10.1073/pnas.1303037110 PMC3657805

[23] Corman VM, Grundhoff A, Baechlein C, Fischer N, Gmyl A, et al (2015) Highly divergent hepaciviruses from African cattle. J Virol 89: 5876–5882. doi: 10.1128/JVI.00393-15 25787289 10.1128/JVI.00393-15 PMC4442428

[24] Baechlein C, Fischer N, Grundhoff A, Alawi M, Indenbirken D, et al (2015) Identification of a Novel Hepacivirus in Domestic Cattle from Germany. J Virol 89: 7007–7015. doi: 10.1128/JVI.00534-15 25926652 10.1128/JVI.00534-15 PMC4473572

[25] Lauck M, Sibley SD, Lara J, Purdy MA, Khudyakov Y, et al (2013) A novel hepacivirus with an unusually long and intrinsically disordered NS5A protein in a wild Old World primate. J Virol 87: 8971–8981. doi: 10.1128/JVI.00888-13 23740998 10.1128/JVI.00888-13 PMC3754081

[26] Scheel TK, Simmonds P, Kapoor A (2015) Surveying the global virome: identification and characterization of HCV-related animal hepaciviruses. Antiviral Res 115: 83–93. doi: 10.1016/j.antiviral.2014.12.014 25545071 10.1016/j.antiviral.2014.12.014 PMC5081135

[27] Thézé J, Lowes S, Parker J, Pybus OG (2015) Evolutionary and Phylogenetic Analysis of the Hepaciviruses and Pegiviruses. Genome Biol Evol 7: 2996–3008. doi: 10.1093/gbe/evv202 26494702 10.1093/gbe/evv202 PMC5635594

[28] Scheel TK, Kapoor A, Nishiuchi E, Brock KV, Yu Y, et al (2015) Characterization of nonprimate hepacivirus and construction of a functional molecular clone. Proc Natl Acad Sci U S A 112: 2192–2197. doi: 10.1073/pnas.1500265112 25646476 10.1073/pnas.1500265112 PMC4343093

[29] Catanese MT, Loureiro J, Jones CT, Dorner M, Hahn T von, et al (2013) Different requirements for scavenger receptor class B type I in hepatitis C virus cell-free versus cell-to-cell transmission. J Virol 87: 8282–8293. doi: 10.1128/JVI.01102-13 23698298 10.1128/JVI.01102-13 PMC3719822

[30] Moore MJ, Scheel TK, Luna JM, Park CY, Fak JJ, et al (2015) miRNA-target chimeras reveal miRNA 3’-end pairing as a major determinant of Argonaute target specificity. Nat Commun 6: 8864 doi: 10.1038/ncomms9864 26602609 10.1038/ncomms9864 PMC4674787

[31] Li YP, Gottwein JM, Scheel TK, Jensen TB, Bukh J (2011) MicroRNA-122 antagonism against hepatitis C virus genotypes 1–6 and reduced efficacy by host RNA insertion or mutations in the HCV 5’ UTR. Proc Natl Acad Sci U S A 108: 4991–4996. doi: 10.1073/pnas.1016606108 21383155 10.1073/pnas.1016606108 PMC3064388

[32] Bernstein E, Caudy AA, Hammond SM, Hannon GJ (2001) Role for a bidentate ribonuclease in the initiation step of RNA interference. Nature 409: 363–366. doi: 10.1038/35053110 11201747 10.1038/35053110

[33] Merritt WM, Bar-Eli M, Sood AK (2010) The dicey role of Dicer: implications for RNAi therapy. Cancer Res 70: 2571–2574. doi: 10.1158/0008-5472.CAN-09-2536 20179193 10.1158/0008-5472.CAN-09-2536 PMC3170915

[34] Kambara H, Fukuhara T, Shiokawa M, Ono C, Ohara Y, et al (2012) Establishment of a novel permissive cell line for the propagation of hepatitis C virus by expression of microRNA miR122. J Virol 86: 1382–1393. doi: 10.1128/JVI.06242-11 22114337 10.1128/JVI.06242-11 PMC3264374

[35] Narbus CM, Israelow B, Sourisseau M, Michta ML, Hopcraft SE, et al (2011) HepG2 cells expressing microRNA miR-122 support the entire hepatitis C virus life cycle. J Virol 85: 12087–12092. doi: 10.1128/JVI.05843-11 21917968 10.1128/JVI.05843-11 PMC3209320

[36] Fukuhara T, Kambara H, Shiokawa M, Ono C, Katoh H, et al (2012) Expression of microRNA miR-122 facilitates an efficient replication in nonhepatic cells upon infection with hepatitis C virus. J Virol 86: 7918–7933. doi: 10.1128/JVI.00567-12 22593164 10.1128/JVI.00567-12 PMC3421686

[37] Vogt A, Scull MA, Friling T, Horwitz JA, Donovan BM, et al (2013) Recapitulation of the hepatitis C virus life-cycle in engineered murine cell lines. Virology 444: 1–11. doi: 10.1016/j.virol.2013.05.036 23777661 10.1016/j.virol.2013.05.036 PMC3755106

[38] Gao M, Nettles RE, Belema M, Snyder LB, Nguyen VN, et al (2010) Chemical genetics strategy identifies an HCV NS5A inhibitor with a potent clinical effect. Nature 465: 96–100. doi: 10.1038/nature08960 20410884 10.1038/nature08960 PMC7094952

[39] Da Costa D, Turek M, Felmlee DJ, Girardi E, Pfeffer S, et al (2012) Reconstitution of the entire hepatitis C virus life cycle in nonhepatic cells. J Virol 86: 11919–11925. doi: 10.1128/JVI.01066-12 22896615 10.1128/JVI.01066-12 PMC3486316

[40] van der Ree MH, de Vree JM, Stelma F, Willemse S, van der Valk M, et al (2017) Safety, tolerability, and antiviral effect of RG-101 in patients with chronic hepatitis C: a phase 1B, double-blind, randomised controlled trial. The Lancet. doi: 10.1016/S0140-6736(16)31715-9 10.1016/S0140-6736(16)31715-9 28087069

[41] Pfaender S, Brown RJ, Pietschmann T, Steinmann E (2014) Natural reservoirs for homologs of hepatitis C virus. Emerging microbes & infections 3: e21 doi: 10.1038/emi.2014.19 26038514 10.1038/emi.2014.19 PMC3974340

[42] Israelow B, Mullokandov G, Agudo J, Sourisseau M, Bashir A, et al (2014) Hepatitis C virus genetics affects miR-122 requirements and response to miR-122 inhibitors. Nat Commun 5: 5408 doi: 10.1038/ncomms6408 25403145 10.1038/ncomms6408 PMC4236719

[43] Thomson AW, Knolle PA (2010) Antigen-presenting cell function in the tolerogenic liver environment. Nat Rev Immunol 10: 753–766. doi: 10.1038/nri2858 20972472 10.1038/nri2858

[44] Gao B (2016) Basic liver immunology. Cell Mol Immunol 13: 265–266. doi: 10.1038/cmi.2016.09 27041634 10.1038/cmi.2016.09 PMC4856812

[45] Nasheri N, Singaravelu R, Goodmurphy M, Lyn RK, Pezacki JP (2011) Competing roles of microRNA-122 recognition elements in hepatitis C virus RNA. Virology 410: 336–344. doi: 10.1016/j.virol.2010.11.015 21185047 10.1016/j.virol.2010.11.015

[46] Blight KJ, McKeating JA, Rice CM (2002) Highly permissive cell lines for subgenomic and genomic hepatitis C virus RNA replication. J Virol 76: 13001–13014. doi: 10.1128/JVI.76.24.13001-13014.2002 12438626 10.1128/JVI.76.24.13001-13014.2002 PMC136668

[47] Yi Z, Sperzel L, Nürnberger C, Bredenbeek PJ, Lubick KJ, et al (2011) Identification and characterization of the host protein DNAJC14 as a broadly active flavivirus replication modulator. PLoS Pathog 7: e1001255 doi: 10.1371/journal.ppat.1001255 21249176 10.1371/journal.ppat.1001255 PMC3020928

[48] Scheel TKH, Gottwein JM, Mikkelsen LS, Jensen TB, Bukh J (2011) Recombinant HCV variants with NS5A from genotypes 1–7 have different sensitivities to an NS5A inhibitor but not interferon-α. Gastroenterology 140: 1032–1042. doi: 10.1053/j.gastro.2010.11.036 21111742 10.1053/j.gastro.2010.11.036

[49] Lindenbach BD, Evans MJ, Syder AJ, Wölk B, Tellinghuisen TL, et al (2005) Complete replication of hepatitis C virus in cell culture. Science 309: 623–626. doi: 10.1126/science.1114016 15947137 10.1126/science.1114016

[50] Moore MJ, Zhang C, Gantman EC, Mele A, Darnell JC, et al (2014) Mapping Argonaute and conventional RNA-binding protein interactions with RNA at single-nucleotide resolution using HITS-CLIP and CIMS analysis. Nat Protoc 9: 263–293. doi: 10.1038/nprot.2014.012 24407355 10.1038/nprot.2014.012 PMC4156013

[51] Jones CT, Murray CL, Eastman DK, Tassello J, Rice CM (2007) Hepatitis C virus p7 and NS2 proteins are essential for production of infectious virus. J Virol 81: 8374–8383. doi: 10.1128/JVI.00690-07 17537845 10.1128/JVI.00690-07 PMC1951341

[52] Zuker M (2003) Mfold web server for nucleic acid folding and hybridization prediction. Nucleic Acids Res 31: 3406–3415. doi: 10.1093/nar/gkg595 12824337 10.1093/nar/gkg595 PMC169194

