# Correction: miRNA independent hepacivirus variants suggest a strong evolutionary pressure to maintain miR-122 dependence

**DOI:** 10.1371/journal.ppat.1007303

**Published:** 2018-09-17

**Authors:** Yingpu Yu, Troels K. H. Scheel, Joseph M. Luna, Hachung Chung, Eiko Nishiuchi, Margaret A. Scull, Natalia Echeverría, Inna Ricardo-Lax, Amit Kapoor, W. Ian Lipkin, Thomas J. Divers, Douglas F. Antczak, Bud C. Tennant, Charles M. Rice

The tenth author's name is spelled incorrectly. The correct name is: W. Ian Lipkin.
